# Abnormal brain white matter in patients with right trigeminal neuralgia: a diffusion tensor imaging study

**DOI:** 10.1186/s10194-018-0871-1

**Published:** 2018-06-22

**Authors:** Junpeng Liu, Jiajia Zhu, Fei Yuan, Xuejun Zhang, Quan Zhang

**Affiliations:** 1 School of Medical Imaging, Tianjin Medical University, No. 1, Guangdong Road, Hexi District, Tianjin, 300203 China; 2 Department of Radiology, The First Affiliated Hospital of Anhui Medical University, Hefei, China; 3 Department of Radiology, Pingjin Hospital, Logistics University of Chinese People’s Armed Police Forces, No. 220, Chenglin Road, Hedong District, Tianjin, 300162 China

**Keywords:** Trigeminal neuralgia, Neurovascular compression, Magnetic resonance imaging, Tract-based spatial statistics, White matter

## Abstract

**Background:**

Idiopathic or classical trigeminal neuralgia (TN) is a chronic painful condition characterized by intermittent pain attacks. Enough evidence demonstrates classical TN is related to neurovascular compression (NVC) at the trigeminal root entry zone (REZ), but white matter change secondary to TN are not totally known.

**Methods:**

Visual Analogue Scale (VAS) and diffusion tensor imaging were performed on 29 patients with right TN and 35 healthy individuals. Voxel-wise analyses were performed with TBSS using multiple diffusion metrics, including fractional anisotropy (FA), mean diffusivity (MD), axial diffusivity (AD) and radial diffusivity (RD). Group differences in these parameters were compared between right TN patients and controls using TBSS and correlations between the white matter change and disease duration and VAS in right TN patients were assessed. Multiple comparison correction were applied to test significant correlations.

**Results:**

The right TN patients showed significantly lower FA and higher RD in most left white matter (*P* < 0.05, FWE corrected). Moreover, negative correlations were observed between disease duration and the FA values of left corona radiata, genu of corpus callosum, left external capsule and left cerebral peduncle, and between VAS and the FA values of left corona radiata, left external capsule and left cerebral peduncle (*P* < 0.05). Positive correlations were observed for disease duration and the RD values of left corona radiata, right external capsule, left fornix cerebri and left cerebral peduncle, and for VAS and the RD values of left corona radiata and left external capsule (*P* < 0.05). However, once Bonferroni corrections were applied, these correlations were not statistically significant.

**Conclusion:**

These findings suggest that TN selectively impairs widespread white matter, especially contralateral hemisphere, which may be the hallmark of disease severity in TN patients.

**Electronic supplementary material:**

The online version of this article (10.1186/s10194-018-0871-1) contains supplementary material, which is available to authorized users.

## Background

Trigeminal neuralgia (TN) is the most common form of facial neuropathic pain with an annual incidence of 4 to 5 new patients per 100,000 [1]. It is characterized by recurrent episodes of unilateral brief electric shock-like pains localized to the sensory supply areas of trigeminal nerve and has been considered as one of the most serious pains that can experience [2–4]. Idiopathic or classical TN is mainly caused by neurovascular compression of trigeminal nerve at its root entry zone (REZ) and microvascular decompression (MVD) surgery is most effective method for relieving neuralgic pain [3–7]. However, peripheral nerve injury caused by neurovascular compression does not fully explain the persistence of long-term recurrent pain in TN patients [8–11].

Neurovascular compression may result in focal demyelination of the trigeminal nerve at the REZ, which consequently generates ectopic discharges and pathological cross-activation between afferent nerve fibers [12]. And pain ensues. What’s more, this process may lead to central white matter changes and/or higher brain structures and sensitization of neurons [13–15]. Previous studies have mostly concentrated on abnormalities of trigeminal nerve [16], but the nature of assumed nerve abnormalities is not known. Diffusion tensor imaging (DTI) based on magnetic resonance imaging (MRI) has been considered as a useful and effective examination of the trigeminal nerve system in great detail [8, 12, 17].

Previous studies have demonstrated white matter abnormalities due to chronic pain and peripheral nerve damage in the TN patients [8, 16, 18]. The results of these studies are similar. These studies demonstrate significantly decreased FA and increased AD, RD and MD. However, the mechanism of TN affecting brain white matter remains unclear.

In order to further understand the relationship between TN and brain white matter plasticity, we examined white matter microstructural change and correlation of between white matter abnormality and disease duration and pain intensity in patients with classical TN.

## Methods

### Participants

Twenty-nine patients (age range 35–77 years; 20 females and 9 males) with right-sided TN and 35 healthy control subjects (age range 41–74 years; 27 females and 8 males) were selected for this study. Patients were enrolled from Department of Functional Neurosurgery of Pingjin Hospital of Logistics University of Chinese People’s Armed Police Forces (Additional file 1: Table S1) and control subjects by newspaper advertisement. The patients belonged to a consecutive series of patients who had undergone evaluation for MVD surgery between 2014 and 2016. All these patients had a long duration (more than 1 year) complaint of classical TN according to the International Classification of Headache Disorders criteria (third edition) [19] and had high-resolution imaging to exclude secondary causes of TN. Visual analogue scale (VAS) [20] was used to assess pain intensity in the TN patients. All patients were measured during a painful attack and on medications. Exclusion criteria [18] for both the patients and controls were as follows: (1) other headache disorders; (2) chronic pain elsewhere; (3) previous TN operations; (4) untreated hypertension or diabetes mellitus; (5) left-handed; (6) alcohol or illicit drug abuse, or current intake of psychoactive medications; and (7) MRI contraindications, such as claustrophobia and metallic implants or devices in the body. This study was approved by our institutional review board, and written informed consent was obtained from all patients and control subjects.

### MRI data acquisition

DTI data were obtained using a 3.0-T MR scanner (Verio system; Siemens, Erlangen, Germany) with a 12-channel head coil. Comfortable and tight foam padding was used to limit head movement. Diffusion weighted images were obtained using a single-shot echo planar imaging (EPI) sequence. The scanning location was in alignment with the anterior-posterior commissural plane. The integral parallel acquisition technique (iPAT) was used and the acceleration factor was 2, which can decrease image distortion from susceptibility artifacts. Diffusion sensitizing gradients were applied along 64 non-collinear directions (b = 1000 s/mm^2^) together with an acquisition without diffusion weighting (b = 0 s/mm^2^). The imaging parameters were applied as follows: 48 continuous axial slices, slice thickness of 3 mm and no gap, field of view (FOV) = 256 mm × 256 mm, repetition time/echo time (TR/TE) = 8000/95 ms, and matrix size = 128 × 128. The reconstruction matrix was 256 × 256 with a voxel dimension of 1 mm × 1 mm × 3 mm.

### Data preprocessing

All diffusion weighted images were carefully checked by three radiologists to exclude apparent artifacts resulted from instrument malfunction and subject motion. DTI data was preprocessed using FMRIB’s diffusion toolbox (FDT, http://www.fmrib.ox.ac.uk/fsl, FSL 4.0) [21]. First, the eddy current distortions and motion artifacts were corrected by using the affine alignment of each diffusion-weighted image to the image of b = 0 s/mm^2^ in the DTI dataset. Then, non-brain tissue from the average b0 image was removed using the FMRIB’s Brain Extraction Toolbox, BET. The brain mask was applied to the rest of the diffusion-weighted images. Finally, the diffusion tensor was estimated for each voxel using the DTIFIT function via linear regression to derive FA, MD, AD and RD maps.

### Tract-based spatial statistics (TBSS)

The following steps were adopted to perform voxel-wise analysis of whole brain white matter measures using the TBSS package (http://www.fmrib.ox.ac.uk/fsl/tbss/index.html) [22]. All subjects’ FA images were aligned to a template of the averaged FA images (FMRIB-58) in Montreal Neurological Institute (MNI) space using a nonlinear registration algorithm implemented in FNIRT (FMRIB’s nonlinear registration Tool) [23]. After transformation into the MNI space, a mean FA image was generated and thinned to create a mean FA skeleton of white matter tracts. Each subject’s aligned FA images were then projected onto the mean FA skeleton according to filling the mean FA skeleton by FA values, resulting in an alignment-invariant representation of the central trajectory of white matter pathways for all subjects. These FA values were obtained by searching perpendicular to the local skeleton structure for maximum value, which was from the nearest relevant tract center. During the former registration step, this second local coregistration step can alleviate the malalignment of diffusion-weighted images. Next, this process was repeated for each subject’s MD, AD and RD map using the individual registration and projection vectors obtained in the FA nonlinear registration and skeletonization.

### Statistical analyses

Voxel-wise differences in FA, MD, AD and RD values of white matter between TN patients and healthy controls were tested using a permutation-based inference tool by nonparametric statistic (“randomize”, part of FSL) and two-sample t-tests. The mean FA skeleton was applied as a mask (thresholded at a mean FA value of 0.2 to include only major fiber bundles and exclude peripheral tracts with significant intersubject variability), and the number of permutations was set to 5000 to allow robust statistical inference. Age and gender were entered into the analysis as confound regressors. The significance threshold for intergroup differences was *P* < 0.05 after correcting for family wise error (FWE) applying the threshold-free cluster enhancement (TFCE) option by permutation-testing tool in FSL. The white matter tracts were identified using the Johns Hopkins University ICBM-DTI-81 White-Matter Labels provided in the FSL toolbox. In addition, significant white matter clusters were described by their coordinates in MNI convention and by their cluster size.

To study the relationship between clinical variables (disease duration and VAS) and each of the DTI measures, region-of-interest- (ROI-) based correlation analyses was performed by using a partial correlation (*P <* 0.05). Regional ROI masks were created for brain sites using clusters determined by voxel-by-voxel intergroup analysis procedures mentioned above. After the extraction of each ROI, the mean FA, MD, AD or RD value of the ROI were calculated. Finally, the correlations were calculated between the DTI measures of each ROI and disease duration and VAS with age and gender as covariates of no interest. Because so many correlations were run, the Bonferroni correction was applied to correct for multiple correlation comparisons.

The demographic and clinical data were compared between the two groups using independent-sample *t*-test for age and Chi-square test for sex distribution, which was conducted with Statistical Package for the Social Sciences version 22.0 (SPSS, Chicago, Ill, USA). Differences were considered significant when *P* was less than 0.05.

## Results

### Demographic and clinical data

In our study, 35 patients with right-sided TN were enrolled. Due to the abnormal data quality and no surgery, 6 patients were excluded in this study. Therefore, 29 patients (age range 35–77 years; 20 females and 9 males) and 35 healthy control subjects (age range 41–74 years; 27 females and 8 males) were selected for this study. Demographic and clinical characteristics of each group are summarized in Additional file 1: Table S2. The self-reported duration in TN patients was 10.2 ± 9.6 years (range: 1–30 years) and the VAS was 5.9 ± 3.1 (range: 2–10). There were no significant differences (*P >* 0.05) between TN patients and healthy controls in age and gender.

### Comparison of DTI metrics between TN and controls

**Fig. 1:**
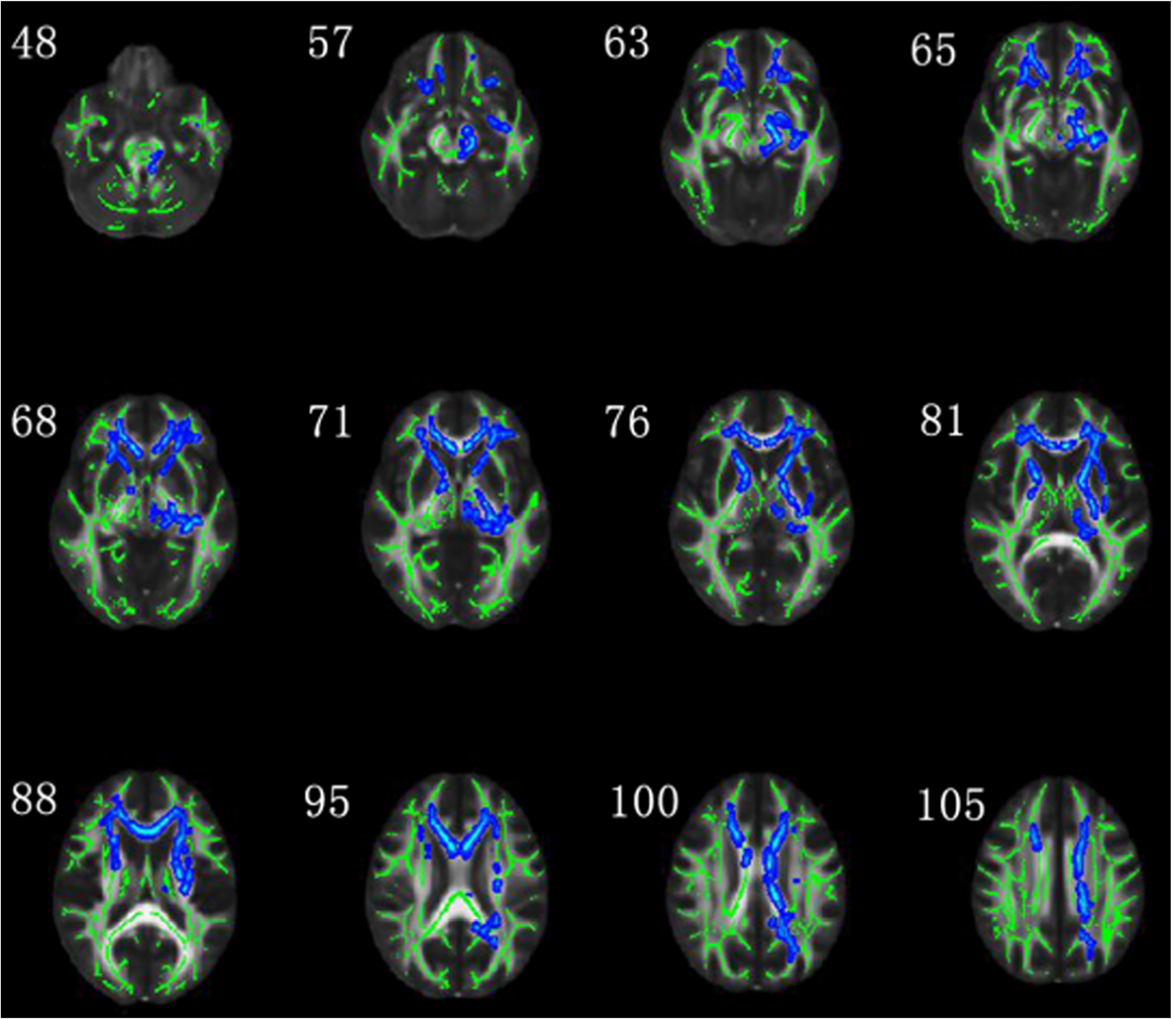
TBSS shows white matter regions with significant differences in FA between TN patients and healthy subjects (*P* < 0.05, FWE corrected). Green represents mean FA skeleton of all participants; blue represents reduction in right TN patients. Coordinates are in millimeters along z axe

Compared with the control group, the TN group showed significantly lower FA in the bilateral superior corona radiata, bilateral anterior corona radiata, body of corpus callosum, splenium of corpus callosum, genu of corpus callosum, left cingulum, left superior fronto-occipital fasciculus, bilateral anterior limb of internal capsule, left posterior limb of internal capsule, left external capsule, left fornix cerebri, internal sagittal stratum and left cerebral peduncle (*P* < 0.05, FWE corrected) (Fig. 1; Additional file 1: Table S3). Moreover, the TN group demonstrated higher RD in the bilateral superior corona radiata, bilateral anterior corona radiata, body of corpus callosum, splenium of corpus callosum, left cingulum, left superior fronto-occipital fasciculus, bilateral anterior limb of internal capsule, bilateral posterior limb of internal capsule, bilateral external capsule, left retrolenticular portion, left fornix cerebri, pontine crossing tract, corticospinal tract and left cerebral peduncle (*P* < 0.05, FWE corrected) (Additional file 1: Figure S1, Table S3). However, no significant difference was found in MD and AD between the TN group and control group.

### Correlations between clinical variables and altered DTI metrics

**Fig. 2:**
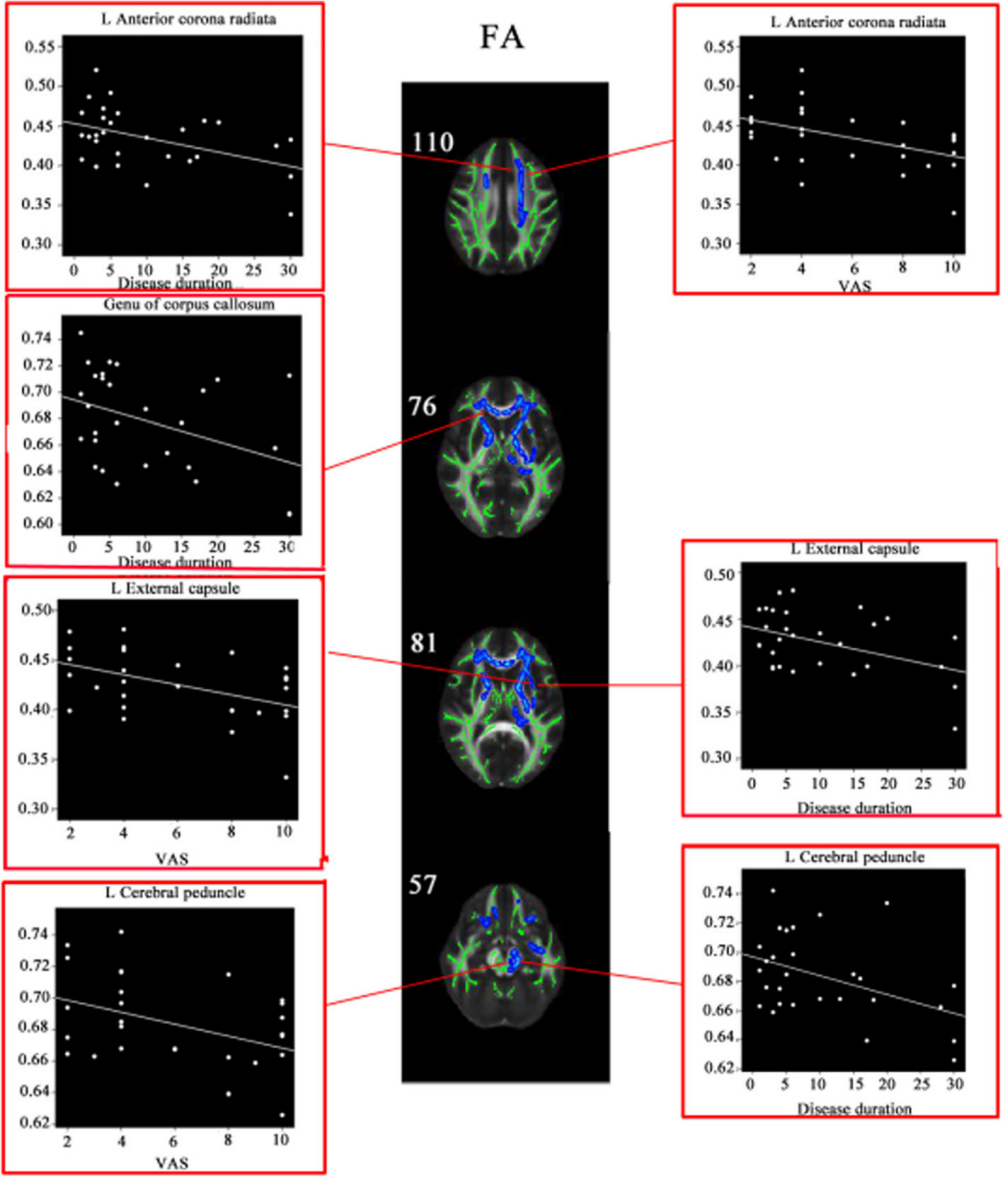
Correlation between the decreased FA and disease duration and VAS. Coordinates are in millimeters along z axe

In the TN patients, negative correlations were observed between disease duration and the FA values of left anterior corona radiata (*r* = 0.211, *P* = 0.012), genu of corpus callosum (*r* = 0.166, *P* = 0.028), left external capsule (*r* = 0.190, *P* = 0.018), left cerebral peduncle (*r* = 0.192, *P* = 0.017), and between VAS and the FA values of left anterior corona radiata (*r* = 0.221, *P* = 0.010), left external capsule (*r* = 0.218, *P* = 0.011), left cerebral peduncle (*r* = 0.168, *P* = 0.027) (Fig. 2). Positive correlations were observed for disease duration and the RD values of left anterior corona radiata (*r* = 0.190, *P* = 0.018), right external capsule (*r* = 0.170, *P* = 0.026), left fornix cerebri (*r* = 0.168, *P* = 0.027), left cerebral peduncle (*r* = 0.156, *P* = 0.034), and for VAS and the RD values of left anterior corona radiata (*r* = 0.174, *P* = 0.025), left external capsule (*r* = 0.191, *P* = 0.018) (Additional file 1: Figure S2). However, once Bonferroni corrections were applied, these correlations were not statistically significant.

## Discussion

### Methological consideration

Compared with voxel-based analysis (VBA), the TBSS method is applied more and more popularly to reveal microstructural alterations of white matter fibers between groups [24–27]. VBA has two severe limitations about different subjects’ alignment of FA images and the self-willed choice of smoothing kernels without any principle or standard [28]. TBSS solve these issues using carefully tuned non-linear registration, and then projecting onto the “mean FA skeleton”(an alignment-invariant tract representation). Moreover, TBSS doesn’t need a smoothing process [22]. Therefore, TBSS can avoid the two limitations and provides us with more diffusion metrics [29]. In many disease studies, TBSS has a wide application to research microstructural white matter alterations, such as adiposity, parkinsonism, Alzheimer’s disease (AD), genetic disease, type2 diabetes mellitus and so on [30–35]. In this study, we have been showed well results of the diffusion metrics (FA, MD, AD and RD) in TBSS methods between groups.

### White matter impairment in TN patients

The primary diffusion metrics (FA and MD) reflect overall white matter health, organization and maturation [36]. In addition, AD reflects axon integrity and RD reflects myelin sheath integrity [31]. Both AD and RD are of great significance in understanding the underlying physiological mechanism [26]. Decreased FA values maybe based on predominantly increase of RD or both RD and AD [30]. The study of DeSouza et al. showed the right-sided TN patients had significantly decreased FA and increased RD, MD, and AD in the brain white matter including the corpus callosum, posterior corona radiata, cingulum, and superior longitudinal fasciculus. Moreover, MD and RD changes of brain white matter in TN patients maybe have relation to central nervous system plasticity, neuroinflammation and edema [18]. In our study, we found the similar results that reduced FA and elevated RD in the corona radiata (mainly concentrating on bilateral superior corona radiate and anterior corona radiata), corpus callosum and left cingulum. Additionally, we found reduced FA and elevated RD in the fronto-occipital fasciculus, internal capsule, external capsule, fornix cerebri and cerebral peduncle in the TN patients. Moreover, altered FA and RD was mainly located in white matter of left hemisphere. However, the differences of MD and AD were not statistically significant. As we all know, TN is involved in trigeminal nerve functional disorders, but other theories of central nervous system pathology is not clear [18, 37]. Due to peripheral trigeminal nerve injury, central nervous system plasticity will most probably occur [18, 38, 39], including fiber organization changes, astrocyte morphology and angiogenesis [18, 40–42]. In our study, compared with healthy controls, TN patients showed decreased FA. The decreased FA may correspond to less fiber organization, such as more axonal sprouting/branching, larger axons, or more crossing fibers [18, 42]. Besides, neuropathic pain is usually related to chronic painful influence on central nervous system. This leads to central sensitization, a process involving demyelination and neuroinflammation processes [18]. The decreased FA is presumably caused by a significant increase of RD, inferring that demyelination and neuroinflammation processes may lead to the impairment of white matter integrity in the TN patients. The RD abnormalities maybe result from these mechanisms in the white matter of TN patients [8].

Many studies have reported cortical and subcortical gray matter impairment in the cognitive-affective, sensory, modulation of pain, attention and motor regions of TN patients [43–45]. In anatomy and/or function respect, these brain areas are connected [13, 46–48]. In our study, we reported decreased FA and increased RD in the corona radiata, corpus callosum, cingulum, fronto-occipital fasciculus, internal capsule, external capsule, fornix cerebri and cerebral peduncle in the TN patients. These fiber connection of brain regions is related to rapid transmission of pain, attention and motor function [49], and maybe lead to the unique sensory symptoms of TN. Our finding also revealed that altered FA and RD was mainly located in white matter of left hemisphere, suggesting contralateral white matter lesions of TN patients.

Correlation analyses found negative correlations between the disease duration and the FA values of left anterior corona radiata, genu of corpus callosum, left external capsule, left cerebral peduncle, indicating the white matter impairment is more and more severe as the disease progressed. With impairing progression of left anterior corona radiata, left external capsule and left cerebral peduncle, painful sensation is more serious. What is more, we infer these regions are probably related to transmission of pain [50]. The RD and the disease duration also reveal positive correlations in the regions of left anterior corona radiata, right external capsule, left fornix cerebri, left cerebral peduncle, demonstrating the white matter demyelination and neuroinflammation of these regions is aggravated in the disease progression. And as demyelination and neuroinflammation of left anterior corona radiate and left external capsule progresses, painful sensation is also more serious. Regrettably, the results of these correlation analyses had not been able to withstand multiple comparison correction.

## Conclusion

In our study, we revealed directly differences between the healthy control and right TN to demonstrate how brain white matter is changed using TBSS methods, suggesting that white matter impairment is the significant hallmark in the right TN. Moreover, the correlation analyses between FA/RD and the disease duration and VAS indicate white matter impairment is more and more severe in the disease progression. And the pain is also more serious with some regions of white matter impairment. The white matter impairment is mostly based on fiber organization, demyelination and neuroinflammation. So we can deeply understand the mechanism of white matter change of TN patients.

### Limitations

Several limitations should be considered when interpreting our results. First, the sample size of the present study was not much, which might cause correlation analyses to fail to withstand multiple comparison correction. Second, non-isotropic voxels were used for DTI data acquisition in this study. In terms of the tensor evaluation, isotropic voxels are more accurate than non-isotropic voxels. Finally, all patients in our study were on medications for TN pain and the anticonvulsant carbamazepine is the most common. The influences of antiepileptics on brain structure are not clear. Future studies are needed to collect more sample sizes, adopt more optimized DTI parameters and avoid the effects of drugs.

## Additional file

Additional file 1: **Table S1.** Characteristics and findings in 29 right TN patients who underwent MVD. **Table S2.** Demographic and clinical data for TN patients and healthy controls. **Table S3.** Comparison of DTI metrics between TN and controls. **Figure S1.** TBSS shows white matter regions with significant differences in RD between TN patients and healthy subjects (*P* < 0.05, FWE corrected). Green represents mean FA skeleton of all participants; red denotes increase in right TN patients. Coordinates are in millimeters along z axe. **Figure S2.** Correlation between the increased RD and disease duration and VAS. Coordinates are in millimeters along z axe. (DOC 1102 kb)

## Abbreviations

AD: Axial diffusivity
DTI: Diffusion tensor imaging
EPI: Echo planar imaging
FA: Fractional anisotropy
FDT: FMRIB’s diffusion toolbox
FOV: Field of view
iPAT: Integral parallel acquisition technique
MD: Mean diffusivity
MNI: Montreal Neurological Institute
MRI: Magnetic resonance imaging
MVD: Microvascular decompression
NVC: Neurovascular compression
RD: Radial diffusivity
REZ: Root entry zone
ROI: Region-of-interest
SPSS: Statistical package for the social sciences
TFCE: Threshold-free cluster enhancement
TN: Trigeminal neuralgia
VAS: Visual analogue scale/score
VBA: Voxel-based analysis
