# Supplementary material for: Abnormal brain white matter in patients with right trigeminal neuralgia: a diffusion tensor imaging study
Source: J Headache Pain. 2018 Jun 22;19(1):46. doi: 10.1186/s10194-018-0871-1 (PMC6013416; doi:10.1186/s10194-018-0871-1)
Supplement: Supplementary file 1 — Table S1. Characteristics and findings in 29 right TN patients who underwent MVD. Table S2. Demographic and clinical data for TN patients and healthy controls. Table S3. Comparison of DTI metrics between TN and controls. Figure S1. TBSS shows white matter regions with significant differences in RD between TN patients and healthy subjects (P < 0.05, FWE corrected). Green represents mean FA skeleton of all participants; red denotes increase in right TN patients. Coordinates are in millimeters along z axe. Figure S2. Correlation between the increased RD and disease duration and VAS. Coordinates are in millimeters along z axe. (DOC 1102 kb) [file 10194_2018_871_MOESM1_ESM.doc]

Additional file 1

**Full title: Abnormal** **brain white matter in Patients with Right** **Trigeminal Neuralgia: A Diffusion Tensor Imaging Study**

**Running title: Brain white matter in TN**

**Tables and Figures**

Table S1 Characteristics and findings in 29 right TN patients who underwent MVD

| Patient No. | Age (y)/gender | Duration of symptoms (y) | Distribution of Pain | Offending vessels revealed by MVD | VAS |
| --- | --- | --- | --- | --- | --- |
| 1 | 75/M | 15 | V1, V2 | SCA and SPV branch | 4 |
| 2 | 65/F | 4 | V2 | PICA | 2 |
| 3 | 77/F | 30 | V1, V2, V3 | TPV | 8 |
| 4 | 63/F | 1 | V2 | PICA | 3 |
| 5 | 61/F | 3 | V2 | SCA | 9 |
| 6 | 75/F | 30 | V2, V3 | SCA | 10 |
| 7 | 54/F | 5 | V1, V2, V3 | SCA | 8 |
| 8 | 58/F | 3 | V1, V2 | SCA and SPV | 10 |
| 9 | 54/F | 1 | V1, V2, V3 | SCA | 10 |
| 10 | 74/F | 17 | V1, V2, V3 | SCA and SPV | 8 |
| 11 | 41/M | 4 | V1, V2, V3 | SCA | 4 |
| 12 | 37/F | 5 | V1, V2, V3 | SCA | 4 |
| 13 | 64/M | 16 | V2 | SCA and SPV | 4 |
| 14 | 38/F | 3 | V2, V3 | AICA | 4 |
| 15 | 54/F | 3 | V2, V3 | SCA | 4 |
| 16 | 58/F | 6 | V1 | SCA and SPV | 4 |
| 17 | 35/F | 2 | V2 | SCA | 2 |
| 18 | 60/F | 10 | V2, V3 | AICA and TPV | 2 |
| 19 | 66/F | 13 | V2, V3 | SCA | 6 |
| 20 | 60/M | 20 | V1, V2 | SCA and SPV and TPV branch | 2 |
| 21 | 73/F | 6 | V2 | SCA | 10 |
| 22 | 62/M | 10 | V2 | SCA | 4 |
| 23 | 71/M | 4 | V2, V3 | SCA and TPV | 2 |
| 24 | 68/F | 2 | V1, V2, V3 | SCA | 10 |
| 25 | 57/F | 18 | V2, V3 | AICA | 6 |
| 26 | 41/M | 30 | V2, V3 | SCA | 10 |
| 27 | 60/F | 6 | V2 | SCA | 10 |
| 28 | 54/M | 1 | V2 | SCA | 4 |
| 29 | 68/M | 28 | V1, V2, V3 | SCA and AICA | 8 |

Abbreviations: AICA = anterior inferior cerebellar artery; F = female; M = male; MVD = microvascular decompression; PICA = posterior inferior cerebellar artery; SCA = superior cerebellar artery; SPV = superior petrosal vein; TN = trigeminal neuralgia; TPV = transverse pontine vein; VAS = Visual Analogue Scale.

Table S2. Demographic and clinical data for TN patients and healthy controls.

| Characteristics | TN patients | Healthy controls | *P* value |
| --- | --- | --- | --- |
| Number | 29 | 35 | NA |
| Gender (females/males) | 20/9 | 27/8 | 0.461 |
| Age (mean ± SD; years) | 59.4 ± 1.9 | 57.6 ± 8.5 | 0.175 |
| Duration (mean ± SD; years) | 10.2 ± 9.6 | NA | NA |
| Pain location (V1/V2/V3) | 12/28/16 | NA | NA |
| VAS (mean ± SD) | 5.9 ± 3.1 | NA | NA |

All participants were right-handed, and free from any visible abnormalities on conventional MRI. Abbreviations: NA = not available; TN = trigeminal neuralgia; SD = standard deviation; VAS = Visual Analogue Scale.

**Table S3. Comparison of DTI metrics between TN and controls.**

| white matter clusters | FA (*P* < 0.05) | RD (*P* < 0.05) |
| --- | --- | --- |
|
| bilateral superior corona radiata | lower | higher |
| bilateral anterior corona radiata | lower | higher |
| body of corpus callosum | lower | higher |
| splenium of corpus callosum | lower | higher |
| genu of corpus callosum | lower | - |
| left cingulum | lower | higher |
| left superior fronto-occipital fasciculus | lower | higher |
| bilateral anterior limb of internal capsule | lower | higher |
| left posterior limb of internal capsule | lower | higher |
| right posterior limb of internal capsule | - | higher |
| left external capsule | lower | higher |
| right external capsule | - | higher |
| left retrolenticular portion | - | higher |
| left fornix cerebri | lower | higher |
| pontine crossing tract | - | higher |
| corticospinal tract | - | higher |
| internal sagittal stratum | lower | - |
| left cerebral peduncle | lower | higher |

Abbreviations: DTI = diffusion tensor imaging; FA = fractional anisotropy; RD = radial diffusivity; TN = trigeminal neuralgia.


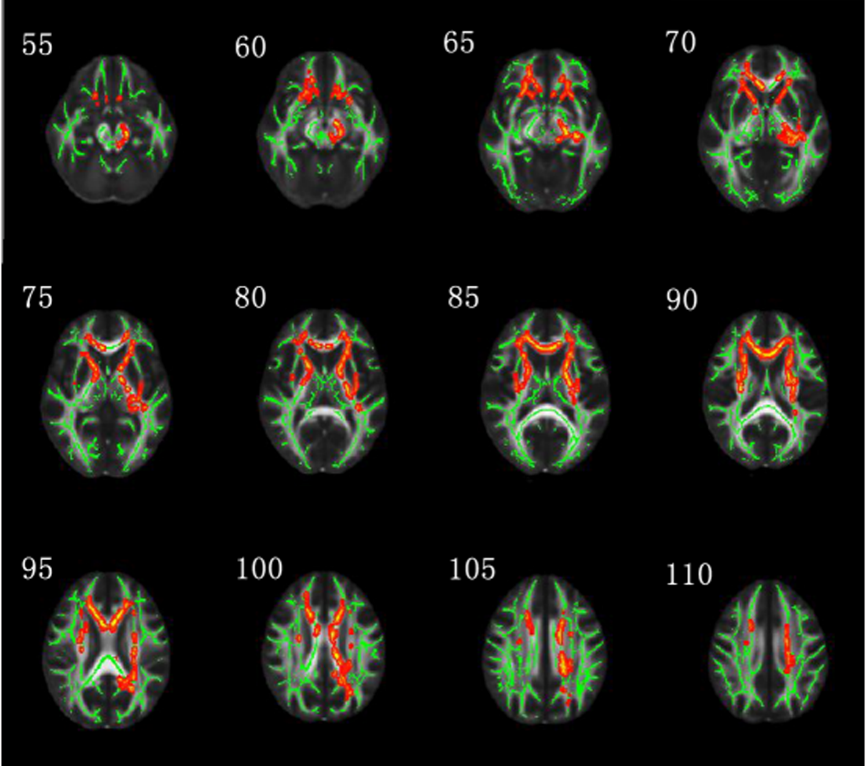


Figure S1 TBSS shows white matter regions with significant differences in RD between TN patients and healthy subjects (*P* < 0.05, FWE corrected). Green represents mean FA skeleton of all participants; red denotes increase in right TN patients. Coordinates are in millimeters along z axe.

**
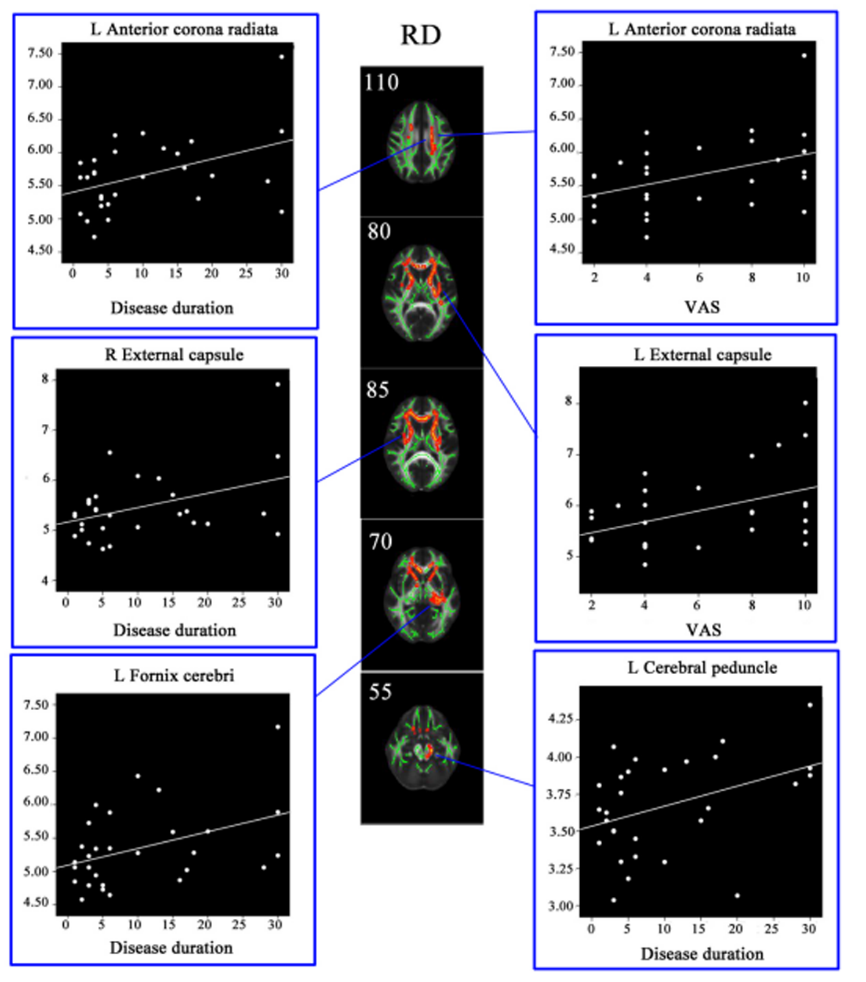
**

Figure S2 Correlation between the increased RD and disease duration and VAS. Coordinates are in millimeters along z axe.
